# Designing for digital transformation of residency education – a post-pandemic pedagogical response

**DOI:** 10.1186/s12909-023-04390-2

**Published:** 2023-06-08

**Authors:** Helena Vallo Hult, Christian Master Östlund, Paul Pålsson, Katarina Jood

**Affiliations:** 1 School of Business, Economics and IT, University West, Trollhättan, Sweden; 2 Department of Planning and Development, NU Hospital Group, Trollhättan, Sweden; 3 Department of Medical Education, NU Hospital Group, Trollhättan, Sweden; 4 Department of Clinical Neuroscience, Institute of Neuroscience and Physiology, Sahlgrenska Academy at University of Gothenburg, Gothenburg, Sweden; 5 Department of Neurology, Sahlgrenska University Hospital, Gothenburg, Sweden

**Keywords:** Physicians, Continuous professional development (CPD), Videoconferencing, Emergency remote teaching (ERT), Workplace learning

## Abstract

**Background:**

The forced transition to emergency remote teaching (ERT) during the COVID-19 pandemic has significantly impacted health professions education worldwide. In Sweden, the need for alternative solutions for the training of junior doctors became urgent, as many of the mandatory onsite courses required for residents to qualify as specialists were canceled. The purpose of this study was to understand course leaders' perceptions and experiences of using digital technologies, such as video conferencing, to teach medical residents (ST) during the pandemic and beyond.

**Methods:**

A qualitative study using semi-structured interviews was conducted with seven course leaders responsible for residency courses during the first year of the pandemic to capture their perceptions and experiences. The interviews were transcribed verbatim and analyzed using thematic analysis, drawing on the technology affordances and constraints theory (TACT) as a framework to explore pedagogical strategies and new teaching practices emerging from the forced use of digital technologies for remote teaching.

**Results:**

The data analysis revealed affordances of, as well as constraints to, teaching specialist medical training during the pandemic. The findings show that the use of digital conference technologies for ERT can both enable and inhibit social interactions, the interactive learning environment and the utilization of technological features, depending on the individual course leaders’ goals of using the technology and the situated context of the teaching.

**Conclusions:**

The study reflects the course leaders' pedagogical response to the pandemic, as remote teaching became the only way to provide residency education. Initially, the sudden shift was perceived as constraining, but over time they found new affordances through the enforced use of digital technology that helped them not only to cope with the transition but also to innovate their pedagogical methods. After a rapid, forced shift from on-site to digital courses, it is crucial to utilize experiences to create better preconditions for digital technology to facilitate learning in the future.

**Supplementary Information:**

The online version contains supplementary material available at 10.1186/s12909-023-04390-2.

## Background

There is a growing body of literature focusing on emergency remote teaching, ERT, during and after the Covid-19 pandemic [1]. A vast majority of the health professions education (HPE) literature on the rapid transition to distance learning during the pandemic has been on undergraduate students in the setting of universities and academic hospitals [2–5]. However, research on professionals' perceptions of digital technology in relation to learning in the workplace is still relatively limited, and there is a lack of research on e-learning for residency education programs in particular [6, 7]. Prior studies have highlighted various ways digital technologies afford and mediate teaching processes and practices, including educational affordances in virtual classrooms [8] and the use and effects of videoconferencing for medical education and professional training in particular [9, 10]. Remote teaching, mediated by videoconferencing, like Zoom, enables flexibility and accessibility to participate from anywhere, and it includes online features such as screen sharing, chat, polls, and virtual group rooms that can facilitate interactive and social learning activities while being physically distant. However, the design process also impacts the quality of the teaching, and differences in the designs of interactive treatment can enable or constrain gains in knowledge and skills [9, 11–13].

Recent studies on ERT and teaching-related practices during times of crisis highlight the importance of viewing remote teaching and learning as a social and cognitive process, which includes the need to support how people learn as they interact and engage with each other and the technology in new ways [13–15]. However, although digital technologies are becoming a core feature of medical work and can support continuing medical education and residency, they are not yet fully integrated into practice [16, 17]. A review of the literature reflects an ambivalence among health professionals, who often respond in contradictory ways when using digital technologies [18]. Previous research highlights individual digital competence, social influence, and meaningful use as important for explaining variations in users' perceptions and desires to assimilate and use digital technology for teaching and learning [19, 20]. In Sweden, the site of this research, pandemic restrictions on public gatherings in early 2020 made it impossible to conduct courses with many people in one place. Consequently, many courses for residents were cancelled [21, 22]. These courses are mostly arranged as onsite courses, face-to-face, and include specialty-specific subjects and general topics required by all specialties, such as leadership, science, communication, ethics and law [23]. Since participation in these courses is mandatory, and a prerequisite for residents to qualify as specialists, a need for alternative solutions for the training of junior doctors quickly arose.

In this study, we adopt a pedagogical perspective that takes its point of departure in socio-cultural learning, stressing that learning occurs through social interactions and collaborations and the importance of learning activities having real-world relevance and utility [24]. We draw on the technology affordance and constraint theory (TACT) [25] to explore pedagogical strategies and new teaching practices emerging from the forced use of digital technologies for remote teaching. Affordances are possibilities, and constraints are challenges to technology use and interaction. This approach aligns with a socio-cultural learning perspective, as it puts forward the notion that the use and outcome of digital technologies are best understood in terms of relationships between people and technology features [25]. It provides a useful framework for our research, as it can help explain why the same technology, in this case, the videoconferencing system (Zoom), may provide different affordances and constraints to different users and over time [26, 27].

### Aim of the study

The overall aim of this study was to understand course leaders' perceptions and experiences of using videoconferencing to teach specialist medical training (ST) during the pandemic. The paper addresses the following research question: What are perceived opportunities and challenges related to emergency remote teaching (ERT) from the perspective of the course leaders? The study contributes with an analysis and increased understanding of how both systems and education can be designed better to take advantage of the opportunities with digital technology.

## Methods

The research approach is qualitative, intending to capture experiences and perceptions and explore patterns and themes in the material. The data was collected using semi-structured interviews with course leaders responsible for teaching residency courses during the pandemic.

### Research setting

The research setting is in Swedish public healthcare, at one of the larger non-university hospital groups in the country, consisting of three hospitals and around 5,000 employees that provide healthcare and medical services for 270,000 inhabitants. The hospital group has 200 doctors enlisted in residency programs in various specialties such as general surgery, internal medicine, psychiatry, and anesthesia. The employee training center is responsible for a hospital-wide residency educational program, open to hospital residents as well as primary care residents in the region. The regulatory body responsible for medical accreditation in Sweden is the National Board of Health and Welfare. In addition to a minimum of five years of clinical service, residents need to take a number of compulsory courses, carry out residency training in other specialties and write a scientific paper [23]. Usually, each course is offered on-site, face-to-face, and twice yearly. Due to the pandemic, almost all courses planned for spring 2020 were canceled. All courses offered in autumn 2020 were digital. Course leaders used various digital tools and platforms to teach, and the residents participated using laptops, tablets, and smartphones. The adaptation to digital platforms was entirely done by the course leaders, as no technical support was available at the hospital.

### Study participants

All course leaders responsible for residency courses during 2020, the first year of the pandemic, were invited to participate in an interview about their experiences of teaching a digital course. Participants received written and oral information about the study and were included after informed consent. All seven course leaders agreed to participate in the study. Five were female, and two were male, and they were responsible for leadership, medical education, medical law, medical ethics, and medical science classes. Five of the course leaders primarily worked as physicians, teaching part-time. One course leader was a hospital lawyer, and one was a management consultant. They all had attended shorter courses in pedagogy; however, they did not have formal degrees in pedagogy. All of them had several years of teaching experience, but only one had engaged in digitalized education as a teacher pre-pandemic.

### Data collection and analysis

The interviews lasted around 30–40 min, were based on a semi-structured interview guide (see Additional file 1) and were recorded and transcribed verbatim. The excerpts used in the findings section are translated from original interviews in Swedish and edited for ease of reading but not substantially altered. The interviews were analyzed collaboratively and iteratively through thematic analysis in several steps [28]. An initial analysis was conducted independently by three of the four researchers in the author team, using an inductive approach (data-based) to identify patterns, connections, and relationships in the material. Based on this, a coding protocol was developed and agreed upon to ensure reliability and validity. A secondary analysis was conducted, guided by TACT [22] (theory-based), to identify occurrences in the empirical material, where the participants described affordances and constraints of using digital technology for ERT. The final analytical step was to collaboratively summarize the findings, which included a discussion of the themes and refinements to reach a final consensus. The qualitative data analysis software NVivo 12 supported the data analysis process [29].

### Ethics

All methods in this study were carried out in accordance with relevant guidelines and regulations in the Helsinki Declaration on research involving humans. The study protocol was reviewed by the Swedish Ethical Review Authority. According to the assessment, the Ethical Review of Research Involving Humans does not apply to the current study: (Reference number 2021–02247) as no intervention or processing of sensitive personal data was made, according to the Swedish law in §§ 3–4 of the act concerning the Ethical Review of Research Involving Humans (SFS 2003:460). Participants received written and oral information about the study and were included after informed consent.

## Results

The results are presented according to the themes and sub-themes derived from the analysis, as shown in Table 1, summarizing how the participants described perceived affordances and constraints of using digital technologies for teaching residents during the Covid-19 pandemic. The three main themes and corresponding affordances and constraints are elaborated further below with examples (illustrative quotes).

**Table 1:** Summary of main themes and identified affordances and constraints related to ERT

Themes (data-based)	Sub-themes (theory-based)	
	**Affordances**	**Constraints**
*Promoting strategies for socialization and inclusiveness* Relates to social relationships and community building through features for communication and collaboration	• New ways of building and maintaining social relationships and a sense of community (digital ice breakers, getting-to-know-each-other, walk and talk, zoom-coffee) • Increased engagement, social interaction and participation in breakout room discussions • Enhanced collaboration, co-creation of ideas and shared learning within the course leader team (e.g., coworking as Zoom moderators)	• Reduced opportunities for social and informal communication and collaboration in Zoom • Less room for spontaneous questions and reflections (typically posed during breaks) • Harder to obtain informal networking and, subsequently, to foster a culture of mutual trust and openness • Inhibiting the extended tacit purpose of building a foundation for continuous learning and collaboration
*Creating an interactive learning environment* Relates to cognitive abilities such as mindset, focus, motivation, and concentration	• More diverse teaching strategies include mixing lectures, group discussions, individual reflection, and interactive polls and quiz • More focused discussions, easier to moderate breakout rooms • Facilitates course planning, structure and time management • Increased flexibility to record and provide course material before, during and after the courses	• Harder to “get a sense” of group dynamics and course participants • Limits the use of body language and nonverbal communication • Increased cognitive load of video meetings, challenge to stay focused (Zoom fatigue) • Risk for distractions and multitasking (e.g., notifications) • Language barriers reinforced • Harder to notice participants who zone out and do not engage in discussions
*Building the plane while flying it* Relates to technological readiness, such as IT infrastructure, training and support	• Increased development of digital competence and skills through learning by doing • Enhanced teaching methods through problem-solving strategies arising from the urgent need to adapt the course design to the digital format • Potential for hybrid teaching and integration of features for digital teaching to enhance onsite courses • The possibility of teaching from home per se: no risk of infection, less traveling, external guest lecturers and participants	• The technology is offered but without sufficient preconditions to learn it properly • Lack of time and training in digital pedagogy • IT-related frustration due to technical problems (internet, video and sound) • Stressful not being able to trust/control the technology • Legal aspects, security and confidentiality issues • Mandatory IT adds complexity due to many systems, and constant changes to the digital environment

### Theme 1: Promoting strategies for socialization and inclusiveness

Firstly, it was notable that the social aspects of ERT were considered a main challenge. Social relationships and physical meeting spaces were highlighted as especially important to the residency courses, as they are primarily based on group discussions and active engagement. While remote teaching was perceived as a social constraint in many ways, the respondents recognized over time that it also had benefits in terms of enabling social elements and community, which could be incorporated into their established teaching practices.

The respondents repeatedly mentioned constrained opportunities for social and informal communication as a downside of remote teaching compared to meeting face-to-face. It was, for example, perceived as more challenging to be available for follow-up questions and spontaneous socializing in Zoom: *“Spontaneous questions and discussions arise more easily face-to-face* (R6). Missing out on small talks and reflections that typically happen between and after lectures were also described as a constraint due to the abrupt ‘end meeting for all’ feature in Zoom: *“It ends, and you are not available anymore, just like that”* (R3). Furthermore, the interviews revealed that the residency program afforded an extended tacit purpose of providing opportunities for informal communication and networking, which builds a foundation for continuous workplace learning and collaboration in clinical practice: *“to actually get to know each other, across clinical wards, which then lowers the threshold when you need each other in practice*" (R1)*.* Something that was perceived as more challenging in the digital courses, especially if the course participants didn’t know each other beforehand: *“That something, which is difficult to explain, but which still all know […] something happens when you meet face-to-face […] which is not possible online. Not as fast, anyway”* (R6).

On the other hand, some respondents described how the social constraint of ERT had become an incentive for developing new forms of interaction and engagement, such as digital ice breakers and getting-to-know-each-other activities, for community building and belonging:*”You have to put much effort into it in the digital format—more than when meeting onsite.”* (R5). Using breakout rooms for discussions along with “digital coffee sessions,” i.e., informal coffee breaks where participants stayed online to chat, provided affordances in terms of a stronger sense of community, more dynamic and engaged discussions and the added value of a more intimate and informal environment: “*They felt that they could sit and talk to other colleagues even though they were not in the same place”* (R1).

Social relationships, collaborative engagement and shared learning also emerged within the course leader team. Respondents emphasized the benefit of having another course leader as a moderator during remote teaching so that one provides the lecture (content) while the other manages the features to support social interaction activities: “…*we had one of the course leaders who were in the chat all the time and collected questions and tried to organize them.* (R4). Conversely, one of the respondents, who was solely responsible for one of the courses, described this as the most significant challenge: *The disadvantage I see is the interaction with the participants, which becomes much more a monologue than a dialogue* [6]. The shared responsibility was not only a matter of sharing the administrative burden. It was addressed as a social aspect, where team collaboration and sharing of experiences was perceived as a key enabling factor for managing the transition.

In summary, this theme illustrates a transition from silo teaching to strategies for socialization and inclusiveness. In-person networking was deemed a core, integrated part of the residency program, not easily translated into the digital setting; but over time, they started exploring, problematizing, and finding novel ways to utilize the new digital technologies and create an inclusive, social learning environment online, beyond replicating traditional teaching methods.

### Theme 2: Creating an interactive learning environment

Secondly, the respondents described the increased cognitive load in the learning environment as demanding, especially initially when many insecurities were combined with the added clinical workload due to the pandemic. Adapting the pedagogy to the digital format, including features for keeping focus, attention, motivation, and concentration, was perceived as both enabling and constraining for teaching purposes.

The course leaders commented on difficulties regarding the lack of direct response in lectures, which *“makes it hard to get a sense of whether course participants are following along and keeping the red thread as a lecturer”* (R3). Being unable to use body language and non-verbal communication was also a challenge. One respondent described how he uses body language to evoke a stressful situation that closer emulates that of an emergency room: *"This is harder to do digitally, in front of the camera"* (R3). Respondents described that they found it more challenging to keep focus in the digital setting, for the lecturer and course participants alike, and it was perceived as demanding and tedious to talk to "black boxes" on a screen or straight into the presentation: *“You just see your screen and no one else around you, so it's like, you've got used to it that way, but you have not got used to wanting it like this”* (R1)*.* Because the courses were mandatory, participants were expected to have the camera on. However, while the course leaders preferred to see the participants, they expressed awareness and understanding of the increased cognitive load of video meetings: “*As the eyes constantly try to interpret the faces you see, it is very strenuous for the cognitive capacity.*” (R5).

Simultaneously, they showed enthusiasm about the possibilities for increased interactivity enabled by digital technologies and provided examples of how they had adapted the course format and embraced online features for remote teaching available in Zoom: “*You can do quizzes and such, so it becomes more interactive that way […] like a chance to actually break up long lectures with various interactive tests and so"* (R4). The respondents also identified cognitive benefits of using the breakout room feature, describing how group discussions became more focused compared to courses with participants on-site and that participants who may be hesitant to talk in larger groups would be more inclined to ask questions: *"We have had journal clubs, and I think that it worked almost better digitally… better presence and to some extent a better discussion… compared to when we met on-site* (R5)*.* While on the other hand, the course leaders felt constrained in that their presence became more noticed, an interference in the discussions, when the purpose is to observe: *“It becomes almost more obvious that you influence the participants as a course leader when you enter the breakout rooms than if you are there on-site”* (R2). They also reflected on the possibility of participating in discussions more anonymously, where the digital tools enable new forms of interaction: *“…with Mentimeter […] people can ask without it being visible who has asked what question”* (R1).

Moreover, the digital course format was described as affording structure and better planning, which helped with cognitive challenges related to focusing, attention and concentration. The respondents reflected on the forced changes to the course design and their role as teachers during ERT, as overall appreciated and beneficial for the teaching: “*They could watch short film clips in advance, and we started with a quiz, and thus the lectures were shorter, which provided more time for questions and also better, higher quality of the questions I think”* (R4). The respondents mentioned problems with language barriers as an example of a constraint, which was reinforced in the digital setting. It was also perceived as more complex to notice participants who zone out and do not engage in discussions, compared to prior seminars where everybody was participating in person. One of the respondents commented on the contradiction that can arise between the need for social and informal interaction on the one hand and the importance of taking a break from the screen on the other: *“if you adjust it, you do walk-and-talk exercises, for example…go outside…that kind of adjustment rather than sitting and having coffee in front of the screen”* (R6).

In sum, the efforts to create engagement through interactive learning activities—while teaching remotely—were considered beneficial overall for the quality of the courses. While remote teaching in itself was perceived as constraining for the course leaders in their pedagogical role, the same constraints also led to strategies that enabled new teaching practices, such as using polls and quizzes, to create engagement and motivation, which turned out beneficial for developing a robust interactive learning environment long term.

### Theme 3: Building the plane while flying it

Finally, aspects related to technological readiness, such as IT infrastructure, training and support, were perceived as a prerequisite for remote teaching. While the course leaders addressed insufficient training and support as constraints, they also developed and improved digital skills and strategies to enhance teaching methods through learning by doing.

The respondents all gave examples of when technical difficulties and problems were a constraint due to management not providing enough resources or time to learn the digital tools properly, which was considered outside their role and responsibility as course leaders: *“I had more of the pedagogical approach, and focused on that, but did not keep track of the technical aspects”* (R4). Issues like poor internet connection or problems with screen sharing, video or sound were perceived as inconvenient and disruptive for teaching. Respondents generally described that they could handle these situations, but not knowing whether they could trust the technology and predict what might go wrong was constraining, as it creates unnecessary stress and pressure. This, in turn, affected the choice of pedagogical strategies utilized during classes, as it made them more cautious about incorporating technical features: *“If you think about the big things, that's the main concern with Zoom, that it gets stressful if it does not work” (R2).*

Respondents stressed lacking knowledge and skills in digital pedagogy, and they expressed that much responsibility was placed on them as individuals to make things work in terms of ‘learning by doing’: *“I cannot say that we have had specific training, no one has trained us in digital tools…but I still think it has worked well " (R2).* The technology is offered, but there is no education, guidance or information on how it works: “*You need structure, examples and support in digital pedagogy so that you do not have to think about the technical parts and can [focus on making] your teaching as good as possible”* (R6). On the other hand, being forced to find strategies to deal with the situation and adapting the course design to the digital format also made them develop new skills and enhance their teaching. The respondents expressed shared feelings of accomplishment and how, in the end, it had worked out better than expected, given the circumstances. As illustrated by the following concluding quote from one respondent: *“I'm a little inexperienced myself. So that might limit some of it, but on the other hand, I thought it went better than I had expected. I thought it would be harder to interact with the participants”* (R3).

Additionally, aspects such as legality and patient confidentiality in digital communication channels also need consideration. It was perceived as both problematic and facilitating that the systems provided and used within healthcare are procured and thus change regularly. As one respondent described, this adds complexity to an already stressed situation: “*So you knew that there were other platforms, including Zoom, which we thought was easier to work with…and then they procured something new…and that has perhaps been the most challenging”* (R1). On the one hand, it ensures security and privacy issues of the systems in use, while on the other hand, it constrains the flexibility of choosing between other available platforms. The respondents described strategies for coping with perceived technical barriers, such as *preparing and providing instructions and training sessions beforehand (R4).* Several respondents stated that they intended to develop the course inspired by their experiences from remote teaching, for instance, using the flipped classroom approach, by recording lectures for participants to watch before the class starts: *"It's a bit of a balancing act… it's still good to have, like web training or recorded material, for when there is time to sit down and read or watch or something"* (R7). They also mentioned affordances of remote teaching per se: “*as many people actually think that it is very nice to be able to participate from home*” (R1), along with benefits of minimizing the infection risk, less traveling, and increased opportunities to invite external lecturers and allowing course participants to participate part of the course days.

In sum, respondents expressed that the transition to remote teaching had worked well, given the circumstances. The shared experience was that it succeeded beyond expectations. Hence, the pandemic, in a way, was an eye-opener, functioning as a catalyst for the possibilities of using digital technologies for teaching that they would likely not have discovered otherwise.

## Discussion

This study presents a picture of course leaders’ experiences of providing online courses during the Covid-19 pandemic. The findings are illustrative of the defining characteristics of ERT [13]. The respondents described the transition as forced upon them in response to Covid-19 while under pressure, with little time for planning and limited support. While videoconferencing, like Zoom, was widespread in health professions education even before the pandemic, the effect of the Covid-19 restrictions marked a dramatic increase in use, including innovation and change, albeit unplanned and despite unprecedented circumstances [9, 13]. Our findings confirm the importance of individual digital competence beyond technical and computer skills and illustrate the importance of the medical community for technology adoption and use [12, 13] in the specific context of ERT in a medical setting. This study put forward the notion of learning as a dimension of digitalization where the design process in itself is influential for the teaching, depending, for example, on the willingness and ability to incorporate features available in systems like Zoom and, in line with the socio-cultural tradition [24], facilitate social learning, interaction and engagement [9, 30].

Efforts to create an interactive learning environment were central to our findings. Our respondents described a variety of strategies to involve the participants, including group discussions and interactive polls and quizzes. Dividing the course into smaller units using breakout rooms was also used. Similar methods have been described in previous health profession education literature published after the Covid-19 pandemic [5, 19]. Zoom fatigue and the struggle to keep participants engaged have also been addressed n previous research [12]. Strategies for socialization and inclusiveness for social interaction (cf. [24]), such as Zoom-coffee breaks, are also described in a review [4]. One innovative find in our study is the concept of digital “walk and talks,” where participants were given a subject to discuss and instructed to go for an individual walk while discussing the topic on their mobile phones with a peer from the course. The strategy activates the course participants and offers the opportunity for physical exercise. It is perhaps a way to address the previously expressed concern that digital teaching promotes physical inactivity [11].

In line with prior research, our study suggests that digital technology can play an essential role in the new way of learning in itself and support physicians’ continuous training [31, 32]. Common barriers include time, accessibility, skills and attitudes, institutional characteristics, and resource features [33, 34], along with differences in contextual factors such as peer support, leadership and adaptation of social, technological and organizational aspects [35, 36]. Similar to findings from ERT in higher education [15], our results shed light on the transition to remote teaching as a process in which digital technology use and practices evolve and change over time. While technical difficulties and problems were perceived as a constraint, it was often quickly approached, dealt with “at the moment,” and solved without affecting the participants or the class. This likely reflects physicians as a profession who are used to making decisions and solving problems at hand [32]. Consistent with prior research, this study shed light on the potential ripple effects of digital courses, as the course leaders, while teaching medical subjects, develop digital skills and learn from each other [27]. This further highlights that peer experts can play an important role in bridging the need for technical support and guidance where interpersonal skills and processes are transformed into intrapersonal ones [24]. Our findings indicate that pedagogical strategies for learning outcomes related to knowledge and understanding work just as well and sometimes better in digital form, whereas skills and abilities (procedural knowledge) are more challenging to digitalize. Our findings suggest that even though remote teaching cannot entirely substitute face-to-face interaction, hybrid approaches have the potential to combine the advantages of both modes of teaching [14].

From an affordance perspective (TACT), the findings of our study illustrate the dual nature of emergency remote teaching, as digital technologies simultaneously enable and constrain pedagogical strategies and teaching practices. How remote teaching is enabled or constrained by digital technology varies depending on the context and course leaders’ individual goals for using the technology. To give one example, it is illustrative how the moderator role emerged as a way for the participants to make use of the affordances of Zoom despite perceived technological constraints. One course leader involved in two separate courses reflected that digital elements were suitable for one of the courses but not the other. This illustrates that the usefulness is context-dependent. For example, he found the course teaching practical skills, such as emergency procedures, more challenging to transfer to a digital setting, while the lecture-based teaching to some extent, even benefitted from the transfer. In related research, TACT has been used to identify affordances and constraints arising from digital technologies, such as social media and video conferencing, on the organizational, group and individual levels in various settings, including healthcare (cf., 27, 37–39). Our study contributes to the use of TACT in the context of continuing medical education and training from a teaching perspective, as few studies with this approach exist.

The participating course leaders’ perceptions and use of digital technologies differ from traditional models of technology adoption [15], as there were few signs of resistance among the respondents. On the contrary, they described feeling overwhelmed at first, but then they started to engage with the technology as they shifted to remote teaching. This is an interesting finding since digital health technologies are considered especially challenging due to tensions and paradoxes of change and control [18, 40]. The forced ‘digital push ‘ can be conceptualized as a digitalization loop – a change process in which certain elements are digitalized while others are not. This process involves retaining and integrating some of the transformative aspects of the original practice while other elements remain unchanged and are at risk of being lost. From the perspective of educators, we believe that our insights into experiences of the transition are important since research in this area is scarce, and videoconferencing will most likely remain an integrated element in post-pandemic medical education and training [6, 7]. For further research, it would be interesting to explore the course leaders’ perceptions and experiences, especially considering “the hidden curriculum” in the form of informal communication and networks built up during the courses. As highlighted in this study and emphasized in prior research [14, 41, 42], this makes up a foundation for continuous workplace learning and the strengthening of inauguration into the organizational culture, which can be both enabled and constrained in remote teaching and learning. Our findings further underline that remote teaching during the pandemic differs from planned and organized online education as part of the regular curriculum, which would also be interesting to explore.

## Conclusions

In sum, our findings reveal that the course leaders found new affordances through the enforced use of the latest technology that helped them cope with the transition and innovate their pedagogical methods in response to the pandemic, as remote teaching became the only way to provide mandatory residency courses. The analysis also identifies constraints accentuated by uncertainties around remote teaching and a perceived lack of competence in digital pedagogy. Drawing on TACT [25] as a theoretical lens, we could identify key benefits and challenges arising from the use of digital technologies in the context of emergency remote teaching (ERT). We thereby provide an increased understanding of how systems and education can be designed better to take advantage of the opportunities with digital technology. Findings from this study are expected to help healthcare education organization make better use of digital tools in education. After a rapid, forced shift from physical courses to digital, it is crucial to utilize experiences to create better preconditions for digital technology to facilitate learning in the future.

### Take home message

The findings from this study can be summarized in the following design considerations for digital educational learning environments within healthcare settings:

- *Use mobile technology to activate participants.*

The course leaders designed activities encouraging participants to walk and talk during group assignments. The digital added value of better learning while doing physical activities.

- *Take into account the cognitive load in digital courses.*

Make adjustments in course design, format and content to avoid zoom fatigue and the cognitive workload of being seated in front of a screen all day.

- *Utilize tools for interactivity.*

To avoid the passiveness that can occur in videoconferencing, various tools can be used to engage the participants, e.g. using polls and quizzes related to the topic of the lecture. The digital format added the value of a higher engagement among participants compared to traditional lectures.

- *Allow the teacher to focus on teaching.*

Use a moderator or co-teacher to manage the digital platform and questions from the participants, to reduce the cognitive load and allow the lecturer to focus on lecturing without disruption.

## Supplementary Information

**Additional file 1.**

## Data Availability

We have full control of all primary data and we agree to allow the journal to review our data if requested. The datasets used and/or analyzed during the current study are available from the corresponding author in response to reasonable requests.
