# Supplementary material for: Designing for digital transformation of residency education – a post-pandemic pedagogical response
Source: BMC Med Educ. 2023 Jun 8;23:421. doi: 10.1186/s12909-023-04390-2 (PMC10248334; doi:10.1186/s12909-023-04390-2)
Supplement: Supplementary file 1 — Additional file 1. [file 12909_2023_4390_MOESM1_ESM.docx]

**Additional file 1: Interview guide**

Questions that will be asked during the interview:

- What was it like to complete the course digitally?
- Were digital tools used during the course? (e.g., Zoom, Skype, Mentimeter, video, etc.)
- How was your experience with the use of technical solutions?
- What advantages and disadvantages do you see with the digital form compared to a face-to-face course?
- Are there course elements that work particularly well in a digital course? If so, what are they?
- Are there course elements that work particularly poorly in a digital course? If so, what are they?
- Is there something in the digital course design that you would like to change next time?
- What is your opinion about courses that blend digital and face-to-face course days?
